# Relationship Between Perceived Stress and Blood Pressure Control in Young Adults With a Family History of Hypertension

**DOI:** 10.7759/cureus.87821

**Published:** 2025-07-13

**Authors:** Junaid Ayaz Khan, Faisal Wali Ahmed, Naveen Shaikh, Ayesha Ghazal Jamali, Jacob George Binoy, Faiz Abdul Rahman, Mavia Habib, Mahbubur Rahman, Zara Jawaid Chaudhry, Aniqa Atiq Mughal, Hamna W Waris

**Affiliations:** 1 Internal Medicine, Sun Yat-sen Medical College, Guangzhou, CHN; 2 Critical Care Medicine, King Saud Medical City, Riyadh, SAU; 3 Epidemiology and Biostatistics, Aga Khan University, Karachi, PAK; 4 Family Medicine, Response Plus Medical Services LLC, Abu Dhabi, ARE; 5 College of Medicine, Liaquat University of Medical and Health Sciences, Jamshoro, PAK; 6 Orthogeriatrics, Cumberland Infirmary, Carlisle, GBR; 7 Geriatrics, Midland Metropolitan University Hospital, Birmingham, GBR; 8 Internal Medicine, Services Hospital, Lahore, PAK; 9 Cardiac Anaesthesia, National Heart Foundation Hospital & Research Institute, Dhaka, BGD; 10 Medicine and Surgery, Bahria University Medical and Dental College, Karachi, PAK; 11 Emergency, Lady Rafat Medical Centre, Karachi, PAK; 12 Medicine, Lumina Research Foundation, Islamabad, PAK

**Keywords:** blood pressure control, cardiovascular health, family history, hypertension, perceived stress, young adults

## Abstract

Background: Hypertension is a primary reason for heart problems that lead to death or illness across the globe, and people with a family history are more prone to it. Stress is known to cause an increase in blood pressure, but the specific influence of stress perception on individuals in this high-risk group remains unclear. This study examines the relationship between perceived stress and blood pressure control in young adults with a family history of hypertension.

Methods: This study employed a cross-sectional design, involving 385 young adults aged 18-35 years from universities, communities, and outpatient centres in Islamabad, Pakistan. Participants completed the Perceived Stress Scale (PSS) in conjunction with the Hill-Bone Compliance to High Blood Pressure Therapy Scale (HB-HBP). All data were run through IBM SPSS Version 26 using Pearson correlation, t-tests, ANOVA, and linear regression to examine the relationship between perceived stress and the effectiveness of blood pressure management. The data were gathered from February 2025 to May 2025.

Results: Perceived stress showed a significant gender difference, with males reporting higher levels of stress (t=3.431, p=0.001). It was moderately positively correlated with blood pressure control (r=0.400, p < 0.001). Regression analysis revealed that the greater the stress, the less effectively a person managed their blood pressure (b=0.400, p < 0.001). Individuals adhering to a particular diet had improved control of their blood pressure (t=3.403, p=0.001). Although physical activity and marital status were found to have a statistically significant correlation with stress and blood pressure control (p < 0.05), the higher values were negligible.

Conclusion: It is shown that controlling blood pressure in young adults who have a family history of hypertension can be affected by their perceived stress. Researchers found that stress management may be very helpful in improving the blood pressure of such high-risk patients. More studies are required to find out how stress can lead to hypertension, including emotional and physical/health aspects.

## Introduction

Hypertension is one of the major causes of cardiovascular diseases such as stroke and heart failure, and the risk is expected to increase exponentially as the systolic blood pressure goes up. Normal blood pressure is defined as being below 120/80 mmHg, while values of 130/80 mmHg or higher are considered high [1,2]. It is common for people to develop essential hypertension with age as they grow older, ultimately having a risk of over 90% in developed areas. Despite various treatments, hypertension cannot easily be controlled due to the difficulties related to its genetic, environmental, and physiological causes [3,4].

Hypertension mainly increases the risk of morbidity and mortality due to the damage it does to arteries, like those seen in atherosclerosis, Charcot-Bouchard aneurysms, and fibrinoid necrosis. Since it is essential for malignant hypertension, controlling blood pressure switches the leading cause of death from atherosclerosis [5]. The leading cause of cardiovascular disease across the world is hypertension, impacting over 1.3 billion adults, and its occurrence is greater in lower-income areas. Although medication and other efforts prevent too much of a rise in blood pressure, very few in resource-limited areas are aware of the problem or can control it [6].

Genetics contributes 30-60% to primary hypertension, while the remaining reasons come from environmental causes. An extensive genome-wide association study identified over 500 loci associated with blood pressure traits, confirming its complex yet highly heritable nature [7,8]. Both national and international guidelines often differ in their assessment of hypertension control since rates can go from as little as 17.5% to 84.6%, indicating there is no standard approach and clarification is needed [9].

While stress can sometimes improve people's performance, too much stress causes ongoing health issues and decreases the quality of life [10]. Subjective well-being is lower in people with hypertension and mediates the connection between stress and blood pressure. People who feel more stressed often have higher blood pressure, but having high subjective well-being helps regulate blood pressure [11].

Over time, higher stress levels have been linked to a higher possibility of developing hypertension, so handling stress is essential for prevention. However, some studies report a reverse association, showing that the association between perceived stress and hypertension is unclear and continues to need further research [12,13]. Individuals with hypertension and insomnia report feeling higher stress, greater anxiety, and depression, and are less able to manage their problems than those who do not have insomnia. Looking at trait anxiety and insomnia together indicates that poor sleep and anxiety problems are essential for people with hypertension to manage [14].

Even though stress can affect the heart and blood pressure, the connection between perceived stress and blood pressure control is seldom analyzed in young adults. This age group is often overlooked in hypertension research, despite being in a critical period where early intervention can shape long-term cardiovascular health. Gaining a clearer understanding of this relationship is essential for developing effective prevention strategies. Dealing with stress could be an alterable behavior aimed at improving blood pressure and stopping hypertension in its tracks. The purpose of this research is to examine the relationship between high perceived stress and blood pressure levels, and to recommend effective ways to manage young patients with hypertension.

Hypertension is a significant public health burden, as it is associated with severe cardiovascular outcomes. It is hazardous when young adults have a family history of hypertension, and they are impacted by both nature and nurture. Although the genetic predisposition cannot be changed, a significant environmental factor, such as stress, can be addressed with the help of behavioral and psychological interventions. Chronic stress can stimulate the sympathetic nervous system and hormonal regulation, which are potential causes of high blood pressure. Nonetheless, the relationship between perceived stress, particularly in combination with aspects of lifestyles such as diet and physical activity, and its impact on blood pressure control in this high-risk population has received little examination. This relationship is vital in advancing early intervention approaches with emphasized stress management, and it could eventually enhance the cardiovascular outcomes of young adults with a genetic predisposition to hypertension.

Primary objective

The primary objective of this study is to examine the correlational and predictive relationship between perceived stress and behavioural blood pressure control, as measured by the Hill-Bone Compliance to High Blood Pressure Therapy Scale (HB-HBP), among young adults with a family history of hypertension.

Secondary objectives

Secondary objectives involve examining subgroup differences in perceived stress and blood pressure control by age, gender, dietary habits, physical activity, and marital status. The purpose of these analyses is to determine the behavioral and lifestyle choices that may need to be changed to inform specific interventions in this high-risk group.

## Materials and methods

Study design and methods

The study employed a comprehensive cross-sectional approach to investigate the interplay between perceived stress and blood pressure control in young adults with a family history of hypertension. Participants were carefully selected from a diverse range of settings, including universities, community centers, and outpatient clinics in both public and private sectors in Islamabad, Pakistan. This meticulous approach ensured a representative sample from various socioeconomic backgrounds, encompassing different levels of income, education, and culture. Only individuals aged 18 to 35 with a family history of hypertension were eligible to participate, further enhancing the study's robustness.

Participants completed questionnaires that assessed stress, blood pressure control, and other factors, including age, gender, lifestyle habits, and past medical histories. The participant's choice and level of knowledge determined whether the survey was self-administered or conducted in person by trained interviewers. Blood pressures were measured manually using a conventional aneroid sphygmomanometer and a stethoscope, by the American Heart Association (AHA) guidelines. Measurements were taken in a seated position after a five-minute rest, and the average of two readings was recorded to ensure accuracy [15]. Each participant learned about the study's purpose, and only those who agreed and signed the consent form were included in the study.

Through this method, the study aimed to shed light on how perceived stress might influence blood pressure in young people with a family history of hypertension. The potential impact of these findings is significant, as they could pave the way for future healthcare strategies that proactively address stress and support those at risk from an early stage, inspiring optimism for the future of hypertension management.

Sample size and technique

Since it is not known how many young adults in the general population have a family history of hypertension, the population was assumed to be infinite for this study. The required sample size was estimated using the formula mentioned below:

\[n = \frac{Z^2 \cdot p (1 - p)}{d^2}\]

Here, Z represents the standard for the desired level of confidence, p is the estimate based on existing studies, and d represents the maximum allowable error. This study used a 95% CI by setting Z to 1.96 and making d equal to 0.05. Based on similar research, which found that around half of the people experience high stress or poor blood pressure, a p-value of 0.50 was chosen to obtain the largest sample size possible [16].

Our study rigorously selected participants to ensure a representative sample. The required sample size for the power analysis was 385 individuals. To account for potential non-responses, a larger group was approached. A total of 450 individuals were invited, and 385 completed the survey, meeting the required sample size. Participants were selected from health centers, community screenings, and outpatient clinics using a convenience sampling method, ensuring that only eligible and willing participants were included at the time of data collection.

The eligibility of study participants was determined based on specific inclusion and exclusion criteria, as detailed in Table 1.

**Table 1:** Inclusion and exclusion criteria for study participants

Inclusion Criteria	Exclusion Criteria
Young people aged between 18 and 35 years	People who have been diagnosed with a psychiatric disorder
People who report that their family members have hypertension	Individuals who are using medications to treat anxiety or depression
Willing to give informed consent	Pregnant women
Ability to answer and process the information in the questionnaire or interview	People who have been diagnosed with a secondary form of hypertension
No other chronic illnesses besides hypertension	People whose answers had incomplete or missing information

Data collection tools

Our data collection process was comprehensive, utilizing a questionnaire with three main sections: demographic information, perceived stress, and compliance with blood pressure treatment. Researchers asked a combination of standardized and original questions to ensure the collection of complete and insightful responses. 

Demographic information

The initial section of the survey consisted of simple questions to establish any relationship between the characteristics of the people and their control of blood pressure. The authors specially designed the demographic questionnaire for this study, which involved questions about age, gender, marital status, level of formal education, occupation, and smoking. Through these variables, it was possible to carry out an in-depth characterization of the whole population and subgroups. (Appendix A). 

Perceived Stress Scale

To assess stress experienced by the participants, the Perceived Stress Scale (PSS) created by Sheldon Cohen et al. in 1983 was used. Stress is measured here by looking into how much respondents feel their lives are unpredictable, uncontrollable, and overloaded. The 10-item PSS often asks individuals to recall their experience over the past month, and they provide a rating (five-point scale) ranging from "never" to "very often." The higher the score is from 0 to 40, the more stress the person feels. Both clinical and general populations have used the PSS, and the test consistently shows a high level of internal consistency with Cronbach's alpha values of 0.78 to 0.91. Since it is easy to use and applicable in many areas, it is appropriate for young adults [17]. We received permission to access this scale from the original authors.

HB-HBP

The HB-HBP, adopted by Hill et al. in 2000, was used to determine how much the participants were following the suggested hypertension recommendations. Treatment compliance is focused on three things: using the prescribed medicines, reporting to follow-up visits, and lowering the intake of salt in the diet. All items are scored using a four-point Likert scale ranging from 'none of the time' to 'all of the time,' and lower scores are preferred since they point to better compliance. The HB-HBP shows high reliability since its Cronbach's alpha values are usually between 0.74 and 0.84, and it is effective in a variety of cultural and health contexts. The study showed whether people from families affected by hypertension can properly follow recommendations for controlling their blood pressure [18]. We received permission to access this scale from the original authors.

Procedure

Individuals were invited to join the study after giving their informed consent at university clinics, community places, and outpatient centres. Participants were approached through direct invitations, informational flyers posted at the sites, and announcements made in community gatherings to ensure a diverse and representative sample. In February 2025, data collection began and was completed in May 2025, spanning a total of four months. Participants had the option to complete the questionnaire on their own (self-administered) or with the help of trained team members (interviewer-administered) if they required assistance due to literacy or comprehension challenges. The original English versions of the standardized questionnaires were used. Although the survey was not formally translated, verbal assistance and clarification were provided in the local language as needed. All the answers were made anonymous so that participants' identities remained protected. As a result of this process, the data collected from different backgrounds among young adults was both fair and fully reflective of everyone. It is important to note that although blood pressure readings were taken during the screening process, these values were not included in the statistical analysis. Blood pressure control was evaluated using the HB-HBP, which assesses behavioral and treatment adherence rather than direct BP values. Incomplete or partially filled questionnaires were excluded using list-wise deletion, and only complete responses were included in the final analysis.

Statistical analysis

We carried out data analysis with IBM SPSS Statistics version 26 (IBM Corp.). To describe the participants, we used descriptive statistics such as means, standard deviations, frequencies, and percentages. Both the Kolmogorov-Smirnov and Shapiro-Wilk tests were done to examine whether the data were normal. Pearson's correlation analysis was used to find out how the PSS and the HB-HBP are related. A t-test was used to determine if there were differences in stress score results between the two genders and also between those following strict diets and those who did not. A one-way ANOVA was used to assess changes in PSS and HB-HBP scores among individuals with no physical activity, light exercise, regular exercise, and intense exercise, as well as those who were single, married, or divorced. Using a simple linear regression, the analysis ascertained whether the PSS scores predicted the HB-HBP scores. There were no confounding or covariate variables in this model. Chi-square tests were used to assess associations between categorical variables: (1) age group (18-21, 22-25, 26-30, 31-35) and physical activity level; and (2) marital status and physical activity level. A significance level of p < 0.05 was used for all statistical tests to determine the factors that influence stress and blood pressure levels.

Ethical considerations

All the ethical practices for research with people were followed during the study. Before starting the research, the protocol was checked and supported by the Lumina Research Foundation’s institutional review board in Islamabad (IRB-2025-0099). Because of this approval, the study was able to maintain the values of respecting individuals, serving their welfare, and privacy. Those taking part were presented with complete details about the purpose of the study, how it would be conducted, potential risks, and likely benefits. All individuals involved provided written consent to participate in the survey before their participation. Taking part was up to the volunteers, and they could end their participation without facing any problems. Throughout the research, participants’ information was kept confidential and not shared outside the academic and analytical process.

## Results

Table 2 gives details about the demographic backgrounds of the 385 participants in the study. The group with the most significant number of participants (N=267, 69%) is that aged 18-21, followed by 47 (12%) participants aged 22-25, another 42 (11%) in the 26-30 age range, and 29 (8%) in the range of 31-35. 85% (N=292) of the participants are female, and 15% (N=93) are male. Among the study subjects, there are 202 single people (52%), 111 people who are divorced (29%), and 72 people who are married (19%). Most participants are well educated, as 70% (N=268) completed an undergraduate degree, 18% (N=69) have postgraduate qualifications, and 12% (N=48) have a high school or equivalent diploma.

**Table 2:** Demographic characteristics of participants (N=385) f: Frequency; DASH: Dietary approaches to stop hypertension

Variable	f	%
Age	-	-
18-21 years	267	69
22-25 years	47	12
26-30 years	42	11
31-35 years	29	8
Gender	-	-
Male	93	24
Female	292	76
Marital status	-	-
Single	111	29
Married	72	19
Divorced	202	52
Educational level	-	-
High school or equivalent	48	12
Undergraduate	268	70
Postgraduate	69	18
Employment status	-	-
Student	117	30
Employed	150	39
Self-employed	86	22
Unemployed	32	8
Do you take any medication for blood pressure?	-	-
Yes	233	60
No	152	40
Do you smoke?	-	-
Yes	160	42
No	225	58
How many days per week do you engage in physical activity?	-	-
None	16	4
1-2 days	97	25
3-4 days	230	60
5+ days	42	11
Do you follow a specific diet (e.g., low-salt, DASH, etc.)?	-	-
Yes	313	81
No	72	19

Among the participants, 39% (N=150) are employed, while 30% (N=117) are students, 22% (N=86) are self-employed, and 8% (N=32) are currently unemployed. When it comes to blood pressure, 233 people (60%) are on medication, compared to 152 people (40%) who are not. Regarding smoking, 160 people (42%) smoke, and 225 (58%) do not smoke. For physical exercise, 60% (N=230) work out three-four times each week, 25% (N=97) work out only one-two times weekly, 11% (N=42) exercise regularly five or more days a week, while 4% (N=16) do not engage in physical activity. To conclude, most people (N=313, 81%) adhere to diets such as low-salt and dietary approaches to stop hypertension (DASH), but 19% (N=72) have not chosen a specific diet. It is evident from the demographic information that the majority of participants are female, young, and focused on their health, and most have a high level of education.

Table 3 displays the findings from the tests used to evaluate the normality of the PSS and the HB-HBP. Results of the Kolmogorov-Smirnov and Shapiro-Wilk tests (p < 0.001 and p < 0.010, respectively) point to the PSS’s distribution being far from normality. In a similar way, the HB-HBP shows important results outside of normality, proven by the Kolmogorov-Smirnov and Shapiro-Wilk tests (p < 0.001). It follows from these results that using non-parametric statistical methods would be helpful since the scales do not redistribute according to a standard curve.

**Table 3:** Normality test to check equal distribution among the PSS and the HB-HBP PSS: Perceived Stress Scale; HB-HBP: Hill-Bone Compliance to High Blood Pressure Therapy Scale; df: Degree of freedom Parametric test: p > 0.05; Non-parametric test: p ≤ 0.05 *p < 0.05, **p < 0.01 considered significant

Variables	Kolmogorov-Smirnov			Shapiro-Wilk		
	Statistic	df	p	Statistic	df	p
PSS	0.069	385	<0.001^**^	0.990	385	<0.010^*^
HB-HBP	0.095	385	<0.001^**^	0.982	385	<0.001^**^

**Figure 1:**
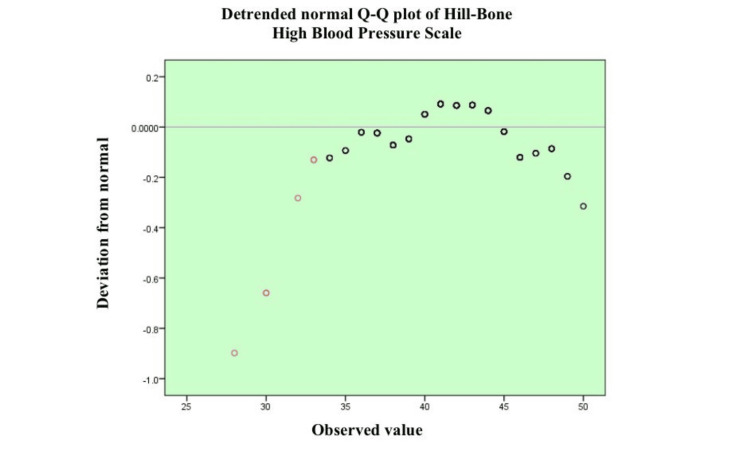
Detrended normal Q-Q plot illustrating the distribution and deviation from normality of HB-HBP scores The Q-Q plot illustrates the deviation of observed values from the expected normal distribution. Each circle represents an individual data point. Black circles denote values that fall within the expected normal range, indicating good adherence to normality. Red circles indicate observed values that significantly deviate from normal expectations, suggesting potential outliers or skewness in the lower tail. The horizontal line at 0 represents perfect agreement with the normal distribution. The shaded green area enhances visual interpretation of deviation magnitude. HB-HBP: Hill-Bone Compliance to High Blood Pressure Therapy Scale

Figure 1 shows a detrended normal Q-Q plot of the observations on the HB-HBP, showing the departure of the values observed to be normally distributed. The majority of the data are close to the zero line, and the data mean values in the center of the distribution are approximately close to normality. Nevertheless, the observed deviations are quite distinct, especially the lower and higher observed values, and, therefore, some form of weak deviations to normality is observed at the extremes of the distribution. There are a few points, at the lower level in particular, that are way below the zero line, which are indicators of possible skewness or the existence of outliers. In general, the distribution is approximately normal. Still, there are some deviations at extremes, so the assumption of normality seems to be not exactly fulfilled and needs to be checked with statistical tests should one intend to perform parametric analysis.

Table 4 shows the correlations between the PSS and the HB-HBP. When using a correlation coefficient of 0.400 (p < 0.001), we notice there is a moderate, positive relationship that is statistically meaningful between the two variables. The findings point to the possibility that people who perceive more stress also tend to have higher scores on the blood pressure scale.

Table 5 shows a comparison of average gender-based scores on the PSS and the HB-HBP. The findings show that there was a significant difference between the perceived stress levels of females (M=27.89, SD=4.58) and males (M=29.71, SD=3.97), where males were scoring higher on the perceived stress (t=3.431, p=0.001), a moderate effect size was found (Cohen's d=0.41). Likewise, males recorded a higher score on the HB-HBP (M=41.37, SD=3.52) compared to females (M=39.00, SD=3.68), and this showed a significant difference (t=3.450, p < 0.001) with a moderate effect size (Cohen's d=0.45). The results indicate that the sample of males under consideration was subjected to a greater amount of stress and was less compliant with the behaviors involving hypertension management than the sample of females. Both dissimilarities were clinically and statistically significant.

**Table 4:** Comparison among variables (gender) PSS: Perceived Stress Scale; HB-HBP: Hill-Bone Compliance to High Blood Pressure Therapy Scale; M: Mean; LL: Lower limit; UL: Upper limitIndependent t-test; **: p < 0.01 considered significant

Variable	Male (N=93); M±SD	Female (N=292); M±SD	t	p	Cl 95% LL	UL	Cohen’s d
PSS	29.71±3.97	27.89±4.58	3.431	0.001^**^	0.775	2.856	0.41
HB-HBP	41.37±3.52	39.00±3.68	3.450	<0.001^**^	1.026	3.574	0.45

Table 6 shows a comparison of perceived stress and managing hypertension behavior amongst individuals who follow a certain diet and those who do not. There is no statistically significant difference between the groups regarding perceived stress levels (p=0.601, Cohen's d=0.04), indicating that perceived stress levels did not correlate with dietary habits. Nevertheless, people following a particular diet exhibited much improved hypertension management behavior, indicated by a higher result of the HB-HBP (p=0.001, Cohen's d=0.44). This implies that dietary alteration has the potential of being used in enhancing compliance to hypertension regimens.

**Table 5:** Comparison among variables (specific diet) PSS: Perceived Stress Scale; HB-HBP: Hill-Bone Compliance to High Blood Pressure Therapy Scale; M: Mean; LL; Lower limit; UL: Upper limit Independent t-test; **: p < 0.01 considered significant

Variable	Yes (N=313); M±SD	No (N=72); M±SD	t	P	Cl 95% LL	UL	Cohen’s d
PSS	28.27±4.56	28.58±4.29	-0.523	0.601	-1.468	0.851	0.04
HB-HBP	41.75±3.49	40.15±4.02	3.403	0.001^**^	0.675	2.521	0.44

Table 7 highlights the comparison between the PSS and the HB-HBP for people who don't exercise, work out one-two days a week, work out three-four days a week, and work out five or more days per week. The PSS results do not indicate any significant changes in the scores between people with different levels of physical activity. Although the p-value is 0.003, which is significant, the analysis shows that there was a slight change in physical activity and perceived stress, suggesting it is not too important. The HB-HBP shows that physical activity plays a significant role in blood pressure scores, as the p-value of 0.023 is below 0.05. Since an F-ratio of 3.209 and an η² value of 0.025 show a small effect size, it means that total physical activity weekly influences blood pressure only a little.

**Table 6:** Comparison of variables (physical activity) PSS: Perceived Stress Scale; HB-HBP: Hill-Bone Compliance to High Blood Pressure Therapy Scale; M: Mean; F: Ratio of variance between groups to within groups; η^2^: Effect size One-way ANOVA; *: p < 0.05; **: p <0.01 considered significant

Variable	None (N=16); M±SD	1-2 days (N=97); M±SD	3-4 days (N=230); M±SD	5+ days (N=42); M±SD	p	F (3,381)	η^2^
PSS	28.38±5.48	28.07±4.29	28.35±4.60	28.83±4.14	0.003^**^	0.281	0.002
HB-HBP	39.44±3.44	40.95±3.65	41.66±3.67	42.24±3.21	0.023^*^	3.209	0.025

Table 8 shows PSS and HB-HBP scores for single, married, and divorced people. An analysis of the PSS results shows that there is a significant difference between people with different marital statuses (p=0.035). For the group that is single, their mean score is 28.62; for married groups, it is 29.33; and divorced groups have the lowest mean score of 27.82. Since the F-ratio turned out to be 3.368 and η² was 0.017, marital status appears to have a weak, but still valid, effect on how much stress a person reports. Also, the results for the HB-HBP demonstrate that there are significant differences between marital groups (p=0.011). Married group has the lowest mean (41.04, SD=3.69), followed by the divorced group (41.37, SD=3.68), and the single group has the highest mean (41.86, SD=3.53). Although the small F-ratio and η² value show that marital status contributes only very little to blood pressure scores in our sample.

**Table 7:** Comparison of variables (marital status) PSS: Perceived Stress Scale; HB-HBP: Hill-Bone Compliance to High Blood Pressure Therapy Scale; M: Mean; F: Ratio of variance between groups to within groups; η^2^: Effect size One-way ANOVA; *: p < 0.05 considered significant

Variable	Single (N=111); M±SD	Married (N=72); M±SD	Divorced (N=202); M±SD	p	F (2,382)	η^2^
PSS	28.62±4.57	29.33±4.33	27.82 4.477	0.035^*^	3.368	0.017
HB-HBP	41.86±3.53	41.04±3.69	41.37 3.681	0.011^*^	1.221	0.006

Table 9 reveals that perceived stress can predict hypertension self-management behaviors to a significant degree, based on an examination of the HB-HBP. The results of the analysis indicated that because of every unit that the PSS increases, the HB-HBP score rises by 1.35 points (B=1.350, 0.400, p < 0.001). The statistical significance of the finding was also confirmed, as the 95% CI (0.547 to 2.153) did not intersect zero, indicating that the finding was statistically significant. This implies that when the stress levels are higher, there is low compliance with practices of managing hypertension.

**Table 8:** Regression analysis included the PSS score as the sole independent variable predicting HB-HBP PSS: Perceived Stress Scale; HB-HBP: Hill-Bone Compliance to High Blood Pressure Therapy Scale; B: Coefficient; SE: Standard error; β: Standardized coefficient; LL: Lower limit; UL: Upper limit **: p < 0.01 considered significant

Variable	B	95% Cl LL	UL	SE	β	P
Constant	41.361	39.031	43.690	1.185	-	<0.001^**^
PSS	1.350	0.547	2.153	0.410	0.400	<0.001^**^

Table 10 presents descriptive statistics comparing the frequency of physical activity among the age groups and the marital status of the participants. Participants who exercised three-four times per week were primarily in the 18-21 age range (N=168), followed by a few in other age categories. Few participants aged 26-35 reported no physical activity. A chi-square analysis indicated a statistically significant relationship between age and frequency of physical activity (χ²=13.2, p < 0.001), suggesting that younger participants are more likely to exercise regularly. In terms of marital status, the largest group of those who exercise three to four times a week (N=120) were also divorced individuals, which might mean that individuals within this group vary considerably. Married and single persons were more uniformly spread in terms of activity levels. There was also a considerable relationship between physical activity and marital status (χ²=39, p < 0.001).

**Table 9:** Descriptive statistics of demographic variables (physical activity per week, age, marital status) F:  Frequency; %: Percentage; p: level of significance p-values calculated using the chi-square test; the significance level is set at p < 0.05.

Variables	f	18- 21 years	Age 22-25 years	26-30 years	31-35 years	p	x^2^	Single	Marital status Married	Divorced	p	x^2^
Physical activity	-	-	-	-	-	<0.001	13.2	-	-	-	<0.001^**^	39
None	16	10	3	3	0	-	-	1	5	10	-	-
1-2 days	97	68	11	12	6	-	-	11	24	62	-	-
3-4 days	230	168	24	21	17	-	-	75	35	120	-	-
5+ days	42	21	9	6	6	-	-	24	8	10	-	-

## Discussion

This research investigated how blood pressure control in young adults is linked to perceived stress if they have a family history of hypertension. Our study found a moderate positive correlation between perceived stress and blood pressure management, suggesting that higher stress levels are associated with poorer blood-pressure control. This aligns with previous research showing that stress can affect cardiovascular function, including heart rate and blood pressure [19]. This association can be described by the biological processes of hypothalamic-pituitary-adrenal (HPA) axis activation and the elevation of cortisol, which both promote blood pressure increase due to the stimulation of sympathetic activity, sodium retention in the kidneys, and gene expression. Individuals at higher risk for hypertension show more persistent cortisol responses to psychological stress [20].

Although we found that males experience greater perceived stress, other studies point out that females react to stress with stronger feelings like anxiety, frustration, and depression. The differences might be due to how people show, experience, or measure stress in the various studies [21]. Our research indicates that men were markedly worse at following up and managing hypertension than women. Likewise, a study also revealed that the level of awareness, treatment, and control of hypertension among younger men was low relative to the same among women, which may be attributed to their low health-seeking behavior and response to treatment [22].

There was no difference in stress levels between people who stick to a particular diet and those who do not, although earlier research shows that there are complex links between diet, worry, and mental health. It may suggest that additional factors are affecting the relationship between variables and should be examined more closely [23]. However, our study found that individuals who adhered to a specific diet demonstrated better blood pressure control, as indicated by HB-HBP scores. These results align with existing evidence that dietary changes, particularly reducing salt intake and increasing consumption of fruits and vegetables, have a positive impact on the management of hypertension. Thus, while diet may not reduce perceived stress, it still plays a vital role in behavioral control of high blood pressure [24].

Our results demonstrated a significant connection between physical activity and perceived stress, but the effect was not significant enough to rely on in practice. However, studies from the past suggest that exercising regularly is linked to lower stress levels, meaning the mental health improvements from working out may depend on factors such as intensity, duration, or the individual’s background [25]. Our research found that having more activity was positively linked to better blood pressure, even if the effect was minimal. This is in line with other studies that confirm that endurance and resistance exercises help reduce blood pressure [26].

In our research, we found a weak but noticeable impact of marital status on stress, with married people showing slightly more stress than those who were divorced. Other research also reveals that stress can fluctuate depending on marital status and is connected to age and education [27]. The study found that married individuals controlled their blood pressure better than those who were single and divorced. The results here are consistent with previous literature that claims that married individuals more often know about their hypertension and can control it [28].

Furthermore, linear regression analysis confirmed that perceived stress was a significant predictor of blood pressure control (β=0.400, p < 0.001). Likewise, studies that monitor physiology indicate that increased stress can lead to heightened blood pressure and heart rate, highlighting the importance for individuals with high blood pressure to recognize and manage stress [19].

Our research identified that younger people were more physically active, and that with age, these activities decreased. This is in line with evidence showing that physical performance begins to decline after 30 years of life due to diminished cardiac output and muscle mass, hence the need to intervene in lifestyle early enough [29]. We found that there is a significant correlation between marital status and physical activity, but divorced people showed the most diverse levels of physical activity. This finding aligns with other general evidence indicating that the effect of marital status on physical activity is age- and gender-dependent, where younger married individuals tend to be less physically active than their unmarried counterparts [30].

Comprehensively, the results of this investigation align well with the hypothesis that perceived stress, diet, physical activity, and marital status are associated with blood pressure control among young adults with a family history of hypertension. Nevertheless, due to the cross-sectional design, it is impossible to establish causation. These findings should inform future studies with longitudinal and interventional designs to determine whether alleviating stress leads to improved hypertension control outcomes.

Limitations

While this study provides valuable information, several drawbacks should be pointed out. With cross-sectional data, it is challenging to establish the cause-and-effect relationship between stress and blood pressure management. Second, because most participants were young, female, and well-educated, the findings may not be applicable to the entire population of young adults with diverse backgrounds. This also means that people’s answers can include bias, resulting from their ability to recall events accurately or from an attempt to present themselves in a favorable light to others. This may have introduced measurement bias, despite the use of validated tools. Since anxiety, depression, and how participants cope with problems were not researched, they may have acted as potential confounding variables, influencing both stress perception and blood pressure control. Moreover, studies that include only those with relatives who have hypertension may mean the results cannot be applied to the rest of the population. Furthermore, it is worth noting that the blood pressure measurements obtained during screening did not appear in the statistical analysis. The control of blood pressure was evaluated behaviorally using the HB-HBP, relative to both systolic and diastolic measures. Although many lifestyle and behavioral risk factors are relevant to hypertension, this study targeted perceived stress, dietary patterns, and physical activity only. Other potential factors, such as smoking, sleep quality, and BMI, were not evaluated and may have influenced the results. Finally, the study did not examine socioeconomic status, which is nonetheless related to both perceived stress and the availability of healthcare for blood pressure management procedures. People with a lower income background can feel more stressed as they are likely to have financial instability and have their ways of treating the disease disrupted, e.g., by the inability to pay for medication or a missed visit. This shortcoming could have influenced how the findings might be generalized to various groups in the economy.

Future directions

Researchers should employ longitudinal approaches in future studies to better elucidate whether high stress causes high blood pressure or vice versa. Studies using interventions should be conducted to determine the effect of stress management programs on adherence to hypertension guidelines. Examining research participants from diverse age groups, genders, socioeconomic backgrounds, and geographic locations would enable the findings to be more broadly applicable to a wider range of people. Also, including extra psychosocial and biological variables such as anxiety, depression, sleep, and measures of stress (cortisol) in studies may give a better explanation of how stress contributes to hypertension. Future studies should also include direct systolic and diastolic blood pressure measurements alongside behavioral scales to assess control more comprehensively. Following these guidelines would help develop approaches that address hypertension in young adults, both in prevention and in its management.

## Conclusions

The results indicated there is a significant relationship between stress and blood pressure control among young adults with a risk of hypertension. However, due to the cross-sectional design, causality cannot be established. While the study supports the idea that psychological factors may play a role in blood pressure management, the findings should be interpreted as correlational rather than causal. Although genetic predisposition cannot be changed, the amount of stress an individual is subjected to is modifiable through behavioural and psychosocial interventions. However, the psychological interventions recommendations may only be offered with precaution and corroborated by prospective longitudinal or interventional studies. The use of stress management techniques will be necessary as routine treatment of hypertension in young individuals with a history of heart disorder in the family to avert the adverse complications of cardiovascular disease in the long term.

## Appendices

Appendix A 

**Table 10:** Demographic information questionnaire DASH: Dietary approaches to stop hypertension

Items	Response
Age	-
Gender	-
Education level	-
Employment status	-
Marital status	-
Do you take any medication for blood pressure?	-
Do you smoke?	-
How many days per week do you engage in physical activity (30 minutes or more)?	-
Do you follow a specific diet (e.g., low-salt, DASH, etc.)?	-
